# A Review of Thermo- and Ultrasound-Responsive Polymeric Systems for Delivery of Chemotherapeutic Agents

**DOI:** 10.3390/polym8100359

**Published:** 2016-10-18

**Authors:** Az-Zamakhshariy Zardad, Yahya Essop Choonara, Lisa Claire du Toit, Pradeep Kumar, Mostafa Mabrouk, Pierre Pavan Demarco Kondiah, Viness Pillay

**Affiliations:** 1 Wits Advanced Drug Delivery Platform Research Unit, Department of Pharmacy and Pharmacology, School of Therapeutic Sciences, Faculty of Health Sciences, University of the Witwatersrand, Johannesburg, 7 York Road, Parktown 2193, South Africa; az-zamakhshariy.zardad@students.wits.ac.za (A.-Z.Z.); yahya.choonara@wits.ac.za (Y.E.C.); lisa.dutoit@wits.ac.za (L.C.d.T.); pradeep.kumar@wits.ac.za (P.K.); pierre.kondiah@wits.ac.za (P.P.D.K.); 2 Refractories, Ceramics and Building Materials, National Research Centre, 33 El-Bohouth St. (former El-Tahrir St.), Dokki, Giza P.O. 12622, Egypt; mostafa.mabrouk@wits.ac.za

**Keywords:** thermoresponsive, ultrasound-responsive, sonoporation, hyperthermia, thermo-ultrasonic responsive, high-intensity focused ultrasound

## Abstract

There has been an exponential increase in research into the development of thermal- and ultrasound-activated delivery systems for cancer therapy. The majority of researchers employ polymer technology that responds to environmental stimuli some of which are physiologically induced such as temperature, pH, as well as electrical impulses, which are considered as internal stimuli. External stimuli include ultrasound, light, laser, and magnetic induction. Biodegradable polymers may possess thermoresponsive and/or ultrasound-responsive properties that can complement cancer therapy through sonoporation and hyperthermia by means of High Intensity Focused Ultrasound (HIFU). Thermoresponsive and other stimuli-responsive polymers employed in drug delivery systems can be activated via ultrasound stimulation. Polyethylene oxide/polypropylene oxide co-block or triblock polymers and polymethacrylates are thermal- and pH-responsive polymer groups, respectively but both have proven to have successful activity and contribution in chemotherapy when exposed to ultrasound stimulation. This review focused on collating thermal- and ultrasound-responsive delivery systems, and combined thermo-ultrasonic responsive systems; and elaborating on the advantages, as well as shortcomings, of these systems in cancer chemotherapy. The mechanisms of these systems are explicated through their physical alteration when exposed to the corresponding stimuli. The properties they possess and the modifications that enhance the mechanism of chemotherapeutic drug delivery from systems are discussed, and the concept of pseudo-ultrasound responsive systems is introduced.

## 1. Introduction

Through the decades of development in cancer treatment, chemotherapy has proven to be the world’s standard management for most cancer conditions [1]. Due to the high cost challenges that arise with new therapeutic approaches, the healthcare fraternity faces difficulty in achieving significant success rates [2]. Escalating efforts are being made by pharmaceutical scientists to advance cancer drug delivery through nanotechnology and the evolution of polymer sciences of biodegradable polymeric nanocarriers. These carriers propose an appropriate approach for transferring chemotherapeutic drugs and proteins in a desirably controlled manner to the target site [3]. Two classes of polymers, namely thermal- and ultrasound-responsive polymers are in many ways associated with advancing cancer therapy [4]. They have also proven to be of value in biomedical applications, ranging from tissue engineering to drug delivery, including gene therapy [5]. Thermoresponsive polymers feature in most copolymeric drug delivery systems [6], with well-known examples including poly(*N*-isopropyl acrylamide) (P(NIPAM)) [5,7], Pluronic F-127 [8] and chitosan [9]. In most cases these thermoresponsive polymers are used to design hydrogels as 3D networks formed by crosslinking of water-soluble polymers [10]. Thermoresponsive hydrogels have been of much interest for decades in the field of drug delivery [11]. Their properties include phase transitioning, altering drug solubility, and controlling drug release at a desired rate [12]. Internal stimulation of smart polymer delivery systems is ideal for drug delivery from a safety perspective [13]; however careful external control of a temperature stimulus is the standard by which thermoresponsive hydrogels respond to modulate drug release [14]. An example of employment of heat as an external stimulus is Hyperthermia Treatment (HT) where accurate and focused heat at temperatures ≥42 °C is produced at specific radio frequencies. The induced heat alters the morphology of the target tissue which assists in increased blood vessel permeability for enhanced drug delivery or ablation of cancerous tissue [15].

Ultrasound is also applied in advanced medical procedures for tumour therapy through heat production. This has proven to be a potently effective and a safe method of tumoral ablation, as well as promoting tissue generation [16]. Ultrasound-responsive polymers can be classified into biodegradable (polylactides, polyglycolides) and non-biodegradable polymers (ethylene vinyl acetate, poly(lactide-*co*-glycolide). Zhou and co-workers [17] suggested the forward-thinking approach of nanocarriers in combination with ultrasound for diagnostic and/or therapeutic applications.

In comparison with other polymeric delivery systems e.g., pH systems, thermal and ultrasound polymer systems can be managed by external stimuli sources such as lasers and MRI guided systems in order to control drug release and delivery desirable for the disease or condition via the ability to tune specific parameters. Whereas pH systems can be designed to respond to pH environmental change but cannot be controlled and managed by external applied stimuli.

In keeping with the fact that these two advanced classes of polymers are commonly applied in chemotherapeutic delivery systems, as well as the potential overlap in their mechanism of stimulation and action, it is pertinent that a concise review of research in this area is assimilated as a foundation of work that has been undertaken, as well as highlighting the potential to achieve advancements in this field. This review provides a concise and critical exploration into the subject matter by discussing the advantages and shortcomings of thermal- and ultrasound-responsive drug delivery in cancer therapy. In addition to the functional properties, phase transitions, as well as underlying scientific mechanisms of these delivery systems are reviewed.

## 2. Advantages and Mechanisms of Cancer Targeting via Thermoresponsive Systems

There are many other advantages that exist with regard to thermoresponsive polymers from their function and design to their application and administration. They are widely used in biomedical applications such as drug delivery and gene therapy, as well as extending to tissue engineering. These smart polymers possess the ability to be developed and transformed into various formulations in order to carry out their function as intended; these include; micelles, hydrogels, particles, and films [18]. These formulations can be modified by adding additional elements such as gold or magnetic material for enhanced performance e.g., good heating properties. External sources of stimulation such as laser power can be used to create the desired response i.e., tumour specificity. Cancer is known to increase normal body temperature [19], augmenting the thermal stimulus towards thermoresponsive systems.

**Figure 1:**
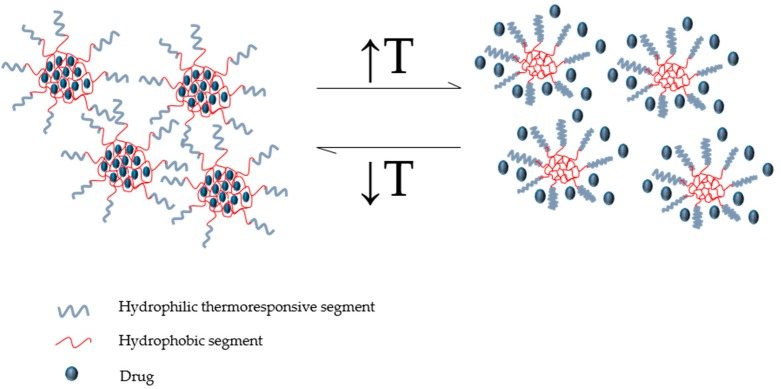
Conceptual illustration representing the drug release mechanism of a thermoresponsive micellar system with a lower critical solution temperature (LCST) (outer shell: thermoresponsive segments, inner core: hydrophobic segments). (Source: de Oliveira et al. [29]. Licenced under a Creative Commons Attribution License).

P(NIPAM) is amphiphilic with a lower critical solution temperature (LCST) between 30–34 °C, often used in cancer chemotherapeutics due to its excellent thermoresponsive behaviour. At room temperature, the P(NIPAM) hydrogel exists as an aqueous gel network. P(NIPAM) hydrogel is generally administered as a depot or an injectable implant exposed to body temperature (37 °C). Hydrogels contain hydrophilic segments which absorb water due to their polarity; the gel network swells as these segments become hydrated leaving the hydrophobic branches of the network exposed to water which in turn causes hydrophobically-bound water. Furthermore, additional absorption of water between the network chains occurs through osmosis [20]. At 32 °C the hydrophilic fragments of the gel network become hydrophobic due to the presence of the isopropyl group present in the polymer and the gel expels the aqueous content [21] from the network, known as a polymer-solvent interaction. These are osmotic mechanisms that are dependent on the polymer-polymer affinity, hydrogen ion pressure and the rubber elasticity of individual strands of the polymer, resulting in shrinkage of the network until all chains within the matrix collapse to form a solidified gel [22]. Once the matrix collapses the drug entrapped within the network is released into the tumour tissue (Figure 1). Various Pluronic^®^ types also function via this mechanism, being used in long-term anti-cancer therapy as demonstrated by Chen and co-workers [23].

P(NIPAM) brushes undergo collapse once it surpasses the LCST. This property allows adsorption of proteins that promotes the adhesion of cells in tissue engineering whereas the swollen brushes cause the opposite extreme below the LCST. There are three mechanisms by which proteins may bind; an attraction between the surface of the brush wall (grafting surface) and the protein (primary adsorption), van der Waals attraction forces between the protein and the surface (secondary adsorption), and protein polymer attractions within the brush. The binding of proteins can be determined by considering the self-consistent field (SCF) theory of brushes to measure the effect of tuning grafting density, degree of polymerization, and the protein dimension parameters [24]. Thermal changes between cell adhesion and cell detachment are based on the hydration state of P(NIPAM) i.e., swelling and osmotic forces. This hydration state is also described with relation to hydrophilic and hydrophobic states but through angle measurement [25].

Several amphiphilic block copolymers also exist as aqueous soluble systems in water at low temperature and have reversible phase transition properties after sol-gel conversion has occurred. This property is known as thermoreversibility, which is composition- and concentration-dependent. Thermogels were produced from polyethylene glycol (PEG)/polyester copolymers via the introduction of hydrophobic blocks onto the copolymer; for example the formation of thermogels has been achieved via introduction of poly(d,l-lactic acid-*co*-glycolic acid) (PLGA), poly(ε-caprolactone) (PCL), poly(ε-caprolactone-*co*-d,l-lactic acid) (PCLA), and poly(ε-caprolactone-*co*-d,l-lactic acid) (PCGA) as the hydrophobic blocks into PEG/polyester copolymers [26]. There is a fine line between the hydrophilic and hydrophobic ratio composition which falls within the viable region to produce a thermoreversible network for cancer therapy application [27]. A PLGA-PEG-PLGA amphiphilic co-polymeric injectable thermogel designed by Ci and co-workers [28] loaded with irinotecan, proved to regress colon tumours through a sustained release mechanism. The team demonstrated that the drug encapsulated within the thermogel provided improved bioavailability compared with drug unaided by the thermogel mechanism [29].

## 3. Advantages and Stimulation Mechanism of Ultrasound-Responsive Polymers in Cancer Therapy

Ultrasound affords a few advantages to drug delivery. As indicated, ultrasound offers more than one mechanism of stimulation i.e., through thermal induction, mechanical stimulus or gas vaporisation through microbubbles. These three mechanisms could also work as an adjunctive stimulus during drug delivery. Due to these multi-mechanisms and the ability to combine them, ultrasound stimulates enhanced permeation of tissues, cell membranes, and specialised barriers, such as the blood brain barrier. In certain cases ultrasound can act as an activator of drugs such as 5-aminolevulinic acid and haematoporphyrin [30], and is also used for guided therapy.

Ultrasound is not only known for its safety and inexpensive diagnostic capabilities through real-time imaging but also for its therapeutic proficiency through focusing high frequencies of ultrasound directed to malignant tumour tissue [31]. The tissue is ablated owing to the thermal, chemical and mechanical effects of High Intensity Focused Ultrasound (HIFU), with minimal side effects. Furthermore, ultrasound is a means of promoting healthy tissue regeneration as synergistic therapy [32]. Recent studies covered in this review highlight that ultrasound in cancer therapy is valuable for chemotherapeutic drug delivery and provides the positive desired outcomes from decreasing tumour growth and size to eradicating tumours completely.

**Figure 2:**
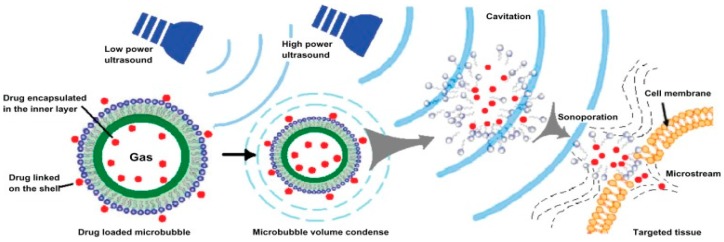
Schematic illustrating low and high power ultrasound assisting in drug delivery though microbubbles and cavitation (Source: Zhao et al. [36]. Licenced under a Creative Commons Attribution License).

Ultrasound plays a role in advancing cancer therapy due to its ability to be easily applied to thermoresponsive systems producing a dual functioning for cancer therapy. Ultrasound produces heat as a “secondary stimulus”, by energy vibration through acoustic cavitation initiated by acoustic vibrational waves. This heat is calculated via a collective time period at 43 °C based on Equation (1):

*tR*^(43−*T*)^ (1)

where *t* represents the time of the treatment, *R* is the constant equivalent to 0.25 for temperatures 37–43 °C and 0.5 for temperatures above 43 °C and *T* is the average temperature throughout the treatment [33]; this increases the blood flow in the tumour vasculature. These acoustic waves employ a release mechanism through cavitation which increases the accumulation of chemotherapeutic drug within the site of the tumour. Once the ultrasound activates the polymer, the polymer responds by creating air-filled microbubbles that eventually burst causing temporary pores [34] in the tissue cell membrane at the focal point of application, enhancing tissue permeability [35] which allows increased passive targeting into tissue (Figure 2).

## 4. Overview of the Properties and Functionality of Diverse Thermo- and Ultrasound-Responsive Systems in Cancer Therapy

### 4.1. Properties of Thermoresponsive Polymer Systems: Temperature Ranges at Phase Transitions

The range of LCSTs for thermoresponsive polymers varies significantly. The LCST can be modified by blending various thermoresponsive polymers in order to customise the physicochemical properties of the system. These include the structural density, surface charge, toxicity, and transfection efficiency within cancer cells [37,38]. Copolymerisation of polymers has also been explored to synthesise thermoresponsive polymers with various LCSTs. Lai and co-workers have co-polymerised a total of ten thermoresponsive polymers with different LCST’s to observe the mechanism by which these polymers changed the 3D structure of a blood clot [39]. Chen and co-workers co-polymerized poly(glycidyl methacrylate) with P(NIPAM) as a pendant to provide a thermo-responsive gating system for the design of nanotubes with a LCST of 32 °C. They also demonstrated that above the LCST (37 °C), the nanotubes remain open sufficiently long enough for the activation of proficient drug release. Below the LCST (25 °C), the gates were in a closing state with no drug release until the activation temperature was once again reached [40].

Optimal cancer therapy focuses on targeted systems for clinical application. Thermoresponsive systems are designed with the LCST responding to the local tumour tissue temperature (~40 °C) that is required for the release of drugs into the cancer cells [41]. A common challenge with thermoresponsive systems is the duration they require to undergo phase transition that results in a burst phase of drug release. However, in recent developments a study has shown that altering the hydrophilic and hydrophobic fragments of the system can enhance the control of drug release by decreasing the thermo-gelling response time [12,42].

Phase transitions in thermoresponsive systems relate to the solubility properties that incorporates the common concepts of LCSTs and upper critical solution temperatures (UCSTs), also known as the cloud point [43,44]. Below the LCST temperature, the structural network of thermoresponsive systems is loosely arranged. As the system is exposed to heat, the network becomes denser until it reaches and surpasses the LCST with solidification at the site of action e.g., solid tumours or cancerous tissue. This subsequently produces an environment for sustained drug delivery, a common goal in cancer chemotherapy.

Colloidal-based thermoresponsive drug delivery systems designed for cancer therapy utilise polymers with a LCST due to the temperature difference between the body and the exterior environment. This facilitates the controlled delivery of the drug to the targeted site. These colloid-based thermoresponsive systems encompass liposomes, micelles, and nanoparticles. Thermoresponsiveness of nanosystems could also occur through thermoreversible swelling via cryotherapy or cold shock, which is used in tumour ablation therapy. This allows increased porosity of the system and promotes drug release from a state of encapsulation [45].

Shape Memory Polymers (SMPs) can provide polymeric systems with thermoreversible properties. These SMPs are frequently conveyed as thermoresponsive polymer systems used in a variety of biomedical applications. At lower temperatures the SMPs are maintained in a specific form. They undergo glass (T*_g_*) and melting (T*_m_*) transitions via the introduction of thermal stimuli greater than the transition temperature that initiates molecular movement. This causes a change in shape of the polymers through formation of crystalline domains (T*_m_*) or an abrupt decrease in the free volume (T*_g_*). After every cooling phase, the SMP system recovers its shape when thermal application is induced. The mechanisms of SMPs and thermoreversability are linked and therefore SMPs may be advantageous to use when designing thermoresponsive systems [46].

There are very few thermoresponsive polymers that have UCST properties. These polymers become soluble at temperatures beyond UCST, whereas LCST polymers become insoluble [47]. Poly(acrylic acid-*co*-acrylamide) (P(AAA)) is a thermoresponsive polymer that possesses an UCST due to hydrogen bonding between the acrylic acid and acrylamide units [18]. The UCST can vary between 2.5–25 °C depending on the ratio of the two monomers. Phase transitions occur due to hydrogen bonding of macromolecules creating intermolecular networks [48]. Unlike most thermoresponsive polymers that work on a negative temperature responsive phase transition system, this polymer functions on a positive temperature responsive phase transition system. A comparison between normal hydrogel and hydrogel nanoparticles produced from P(AAA) was undertaken. The study showed that with an increase in acrylamide, drug entrapment of 5-FU was enhanced in both hydrogel and hydrogel nanoparticles but the nanoparticles were more effective in colon cancer due to a higher concentration of 5-FU reaching the colon [49]. Phase transitions of thermoresponsive hydrogels occur through very specific mechanisms due to the microscopic nature of the gels. These mechanisms are modified in various ways by altering compositions of polymers and by including new functional groups in the system.

### 4.2. Modification of Thermoresponsive System Properties for Enhanced Cancer Therapy

**Figure 3:**
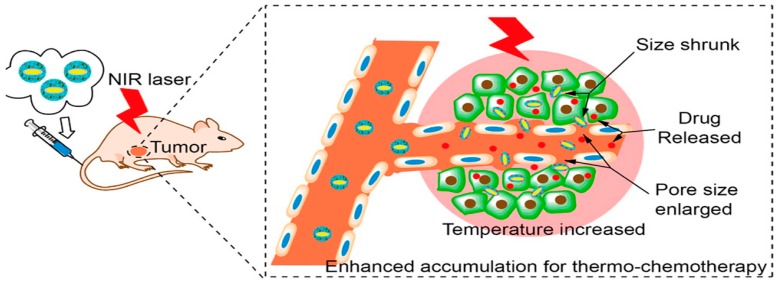
Image illustrating a Near InfraRed Laser being applied to the site of delivery and the mechanism of action that occurs with heat stimulus. Adapted with permission from Zhang et al. [52]. Copyright ^©^ 2014. American Chemical Society.

The physical interaction of polymers with its environment and a change in functional group chemistry are affected by the modification of the polymers conformational state and its solubility during formulation [50]. The most altered and well known property of thermoresponsive polymers is the drastic and reversible changes in solubility [51]. The advantages of using thermoresponsive polymers for cancer therapy are attributed to the mechanisms of targeted chemotherapy. Zhang and co-workers [52] fabricated gold nanorods loaded with doxorubicin for breast cancer which is induced through a thermo-chemotherapy mechanism activated through a Near InfraRed (NIR) Laser at 760 nm, 500 mW, and 16 W/cm^2^ (Figure 3). Their studies showed a higher accumulation of doxorubicin in tumours that were heated compared to those that were not exposed to heat [52].

Several types of thermoresponsive colloidal carriers were designed for optimum use in cancer therapy. These carriers enhance the chemotherapeutic effect by decreasing renal clearance due to increased size, which in turn leads to increased circulation time. Increased circulation times are advantageous when the drug is targeted to function at the site of action to achieve tumour specificity. These colloidal carriers have the ability to extravasate from the tumour vasculature into the tumour tissue, concurrently avoiding entry into healthy tissue and therefore creating a higher quality efficient means of therapy. Furthermore, tumour tissue has a slightly higher temperature than normal tissue and therefore these delivery systems can be designed to respond to tumour temperature [43].

In many drug delivery developments, thermoresponsive polymers are employed as a shell or outer layer with the intention of using heat as the drug release stimulus. In a development by Purushotum and co-workers [53], thermoresponsive core–shell magnetic nanoparticles were designed for the multi-modal treatment of cancer. These magnetic nanoparticles proved to possess good heating properties without the thermoresponsive coat, whereas the coated multifunctional system presented with a significant drug accumulation and release within the tumour and therefore proved to be a promising system for enhanced cancer therapy [53].

Photothermal Ablation (PTA) of laser origin applied in cancer therapy, coupled with a doxorubicin-loaded hollow gold nanoshell system, was used to treat breast cancer. This thermally-responsive drug release was initiated by a 3 W laser that produced temperatures between 54–55 °C. However, temperatures between 40–45 °C are said to cause extensive protein denaturation [54]. These temperatures are known to be high enough to cause cell death and tumoral ablation [55] (Table 1). This combinatory mechanism has proven to be efficient through histological analysis; tumour necrosis was >60% in mice that were treated in conjunction with laser treatment compared to mice without laser application (<10%). In evaluating the above systems, the variety of stimulating methods requires an understanding of the applicable chemistry that gives rise to specific properties through functional structures and trends in polymer sciences.

Thermoresponsive polymers vary in properties according to the synthesis of different copolymer blends, as well as side-chain or element attachment to existing polymers to produce a new polymer or derivative. Modifications of methods of synthesis also alter the structure during polymer formation, having an effect on the thermoresponsive ability of the polymer. In a study undertaken by Chen and co-workers [56], di(ethylene glycol) methyl ether methacrylate (DEGMA) and oligo ethylene glycol methyl ether acrylate (OEGA) diblock copolymers were fabricated with a gold nanoparticle core for cancer therapy. It was established that attachment of DEGMA to the gold nanoparticle core had a greater effect in altering the thermoresponsive properties of the system compared to OEGA. Steric bulk and polymeric arrangement towards a metal core caused significant adjustments to the thermoresponsive properties of polymers. Possessing the gold nanoparticle as the core provided value to the systems’ drug release control mechanism by converting light waves of 527 nm to heat for thermoresponsive drug release into tumour tissue and minimal drug diffusion into the blood stream [56].

Synthesising polymeric derivatives also plays a part in altering the responsive properties of a polymer. Kikuchi and co-workers [37] designed four derivatives of a multi-armed star-shaped thermoresponsive delivery system from poly[2-(dimethylamino) ethyl methacrylates] (s-PDMAEMAs). It was concluded that in s-PDMAEMAs with a low degree of polymerisation, a trend of increasing transition temperature was observed [37]. Attaching functionalised groups to a polymer could also change the phase transition temperature. Both phenyl and hydroxyl groups were polymerised terminally to P(NIPAM) for comparative studies. P(NIPAM) with the phenyl termination had a greater effect on lowering the LCST temperature than the P(NIPAM) with hydroxyl termination [57]. Star-shaped amphiphilic co-block polymer micelles were also used in anticancer therapy by Rezaei and co-workers [58]. An innovatively designed paclitaxel-loaded folate-decorated system demonstrated an improved efficacy in terms of delivery and specificity. It has been suggested by Kikuchi et al. [37] that these positive results presented by the micelles promote the application for chemotherapeutic drug delivery to achieve high intracellular uptake and a significant cytotoxic profile in cancer cells [58]. The flexibility of being able to modify thermoresponsive drug delivery systems in these numerous ways allows for greater advantages of these systems in cancer chemotherapeutics.

### 4.3. Advancing Functional Trends in Thermoresponsive Polymers

Structurally, thermoresponsive polymers are known to have methyl, ethyl, and propyl groups [59]. Amide functional groups and amide-amide interactions tend to produce hydrogen bonding and dipole interactions between polymers and allows for thermophilic behaviour [60]. Well-established trends of functionalising thermoresponsive polymers are associated with the attachment of antibodies for passively-targeted specific delivery which leads to longer circulation time and accumulation of chemotherapeutic drugs in the desired pathological areas [61]. Antibody attachment also assists with masking the release of drug without any interference by the body’s natural defence. This is achieved through surface modification of the hydrophobic segment of the carrier system [62].

Introducing thermoresponsive systems, such as P(NIPAM), with novel properties has been extensively researched in cancer therapy [63,64]. Iron oxide nanoparticles with a thermoresponsive copolymer coating were produced with a second set engineered with R11 peptides for prostate cancer targeting. The iron oxide nanoparticles are guided to the desired site of accumulation via a magnetic field. The R11 conjugated nanoparticles proved to have a higher accumulation in the tumour compared to the nanoparticles without the R11 conjugated peptides [55].

Extensive studies have been also been undertaken with the attachment of RGD (tripeptide Arg-Gly-Asp) sequence peptides to modify thermoresponsive polymers particularly P(NIPAM) for cell adhesion and harvesting in tissue engineering. This method of tissue engineering aims at avoiding the use of foetal bovine serum for cell culture. The optimal sites for the attachment of RGD to the P(NIPAM) brush chains grafting surface, where cell adhesion at 37 °C and cell detachment are achieved. These are prepared according to a guideline designed by Helparin and Kroger 2012 [65]. Avoiding the use of foetal bovine serum terminates any chance of contamination of animal disease and interactions with foreign protein bodies [65] and can have enhanced results on cell culture studies.

**Figure 4:**
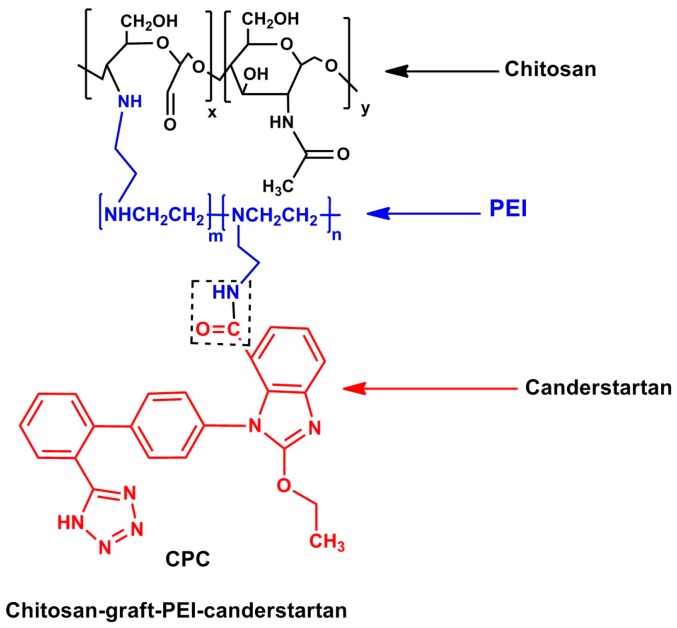
The molecular structure of the Chitosan-graft-PEI-candesartan polymer used in targeting pancreatic cancer cells [66]. (Source: Bao et al., 2014).

Bao and co-workers [66] developed a multi-functionalised chitosan-grafted polyethyleneimine candesartan (CPC) conjugated nano-delivery system (Figure 4.) for use in the treatment of pancreatic cancer. The nanovectors employed multiple mechanisms toward chemotherapy by co-delivery of bafilomycin and the wild type-p53 gene complexed to CPC for the reduction in tumour size. Moreover, the nanovectors hinder tumour growth by promoting anti-angiogenesis via reduction of Vascular Endothelial Growth Factor (VEGF) secretions due to the candesartan. This synchronised approach of hindering tumour growth and reducing tumour size may contribute to elevated success in the development of targeted tumour therapy.

Although many researchers have explored chemotherapeutic delivery systems, brachytherapy, where radioactive seeds or sources are placed in or near the tumour itself for high dose radiation to the tumour and reduced exposure in the surrounding healthy tissues, cannot be ruled out from successful delivery in cancer therapy. A thermally-responsive elastin-like polypeptide that contains a therapeutic conjugated radionuclide has been fabricated by Lui and co-workers [67].

The system is suggested to have a suitable administration route, a well-controlled delivery mechanism and resulted in an increase in survival rate. As observed in the above-mentioned systems, heat can be produced and transferred in more than one way, significantly generating heat for cancer therapy through the exposure of ultrasound. A summative representation of various thermoresponsive systems for cancer therapy is provided in Table 1.

### 4.4. Modification of Ultrasound-Responsive System Properties for Enhanced Cancer Therapy

Microbubbles have become a common trend in cancer therapy used in conjunction with ultrasound therapy. These microbubbles were conventionally used as ultrasound imaging diagnostic tools, demonstrating a positive contribution to the advancement in drug delivery through overcoming limitations of stimuli responsive systems. Numerous delivery systems, as provided in Table 2, employ phospholipid microbubbles such as 1,2-diestearoyl-*sn*-glycerol-3-phosphoethanolamine (DPSE), perfluorocarbon (PFC), perfluoropentane (PFP), 1,2-dietearoyl-*sn*-glycerol-3-phosphotidylcholine (DSPC), and 1,2-dioctadecanoyl-*sn*-glycero-3-phospho-(1′rac-glycerol) (DSPG).

Zhang and co-workers [87] designed methotrexate-loaded nanobubbles composed of the copolymer, poly(lactic acid-*co*-glycolic acid) (PLGA). Both individual units, i.e., poly(lactic acid) and poly(glycolic acid), are classified as ultrasound-responsive polymers [87]. This system was designed for targeted therapy in combination with the use of tumour ablative therapy through HIFU for placental chariocarcinoma. When the combination therapy of antibody, nanobubbles, drug, polymer, and HIFU are employed, the proliferation index is decreased and the apoptosis index increases significantly [87]. It can be deduced that including a nano/microbubble component in the formulation assisted with responsivity to ultrasound stimulation.

In delivery systems fabricated from polymers that are not ultrasound-responsive, ultrasound delivery is achieved through the addition of agents such as Levovist, an ultrasound contrast agent that contains microbubbles [88] or perfluoropentane [89]. Those microbubbles are then coated with the drug-loaded polymer via formation of emulsions to acoustically trigger delivery of chemotherapeutic drugs, for example, chlorambucil [90]. Through a similar mechanism, the drug is delivered with efficiency to the intended site. When the ultrasound is transmitted, it interacts with the PFP and vaporisation occurs, thus causing the formation of microbubbles. As the microbubbles increase in volume and surface area, they facilitate drug release by bursting; causing temporary pores within the desired cell layers releasing the drug into the targeted cancerous tissue [91] as depicted in Figure 2.

Yang and co-workers [92] (Table 2) designed poly(methyl methacrylate) (PMMA)-based nanocapsules infused with temperature-sensitive perfluorohexane (PFH), a well-known ultrasound contrasting agent used to conduct image guided therapy in liver cancer. The nanocapsules were uniformly shaped and arranged suggesting easy diffusion into cancer cells, with a high drug-loading ability of >90%. Following injection, the ultrasound energy caused PFH to form small bubbles creating a strong imaging signal as well as enhancing vascular endothelium permeability. Further, drug release could also be triggered via ultrasound and conditions emanating from glutathione reduction.

**Figure 5:**
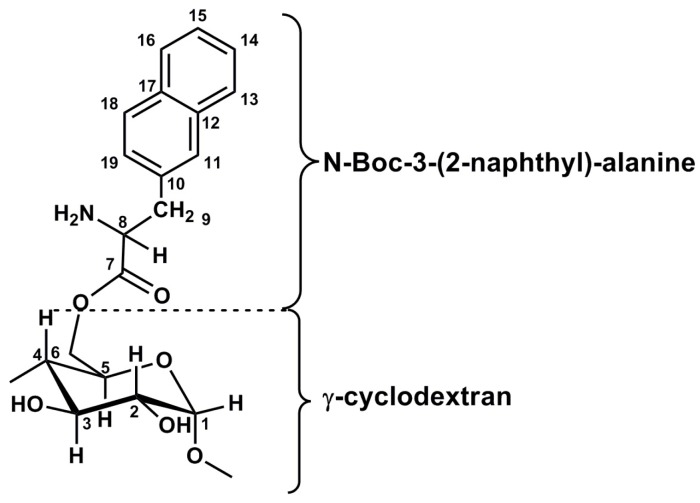
The Molecular structure of the γ-cyclodextran nanocarrier system used against breast cancer cell lines [93]. (Source: Gourevich et al., 2013).

**Figure 6:**
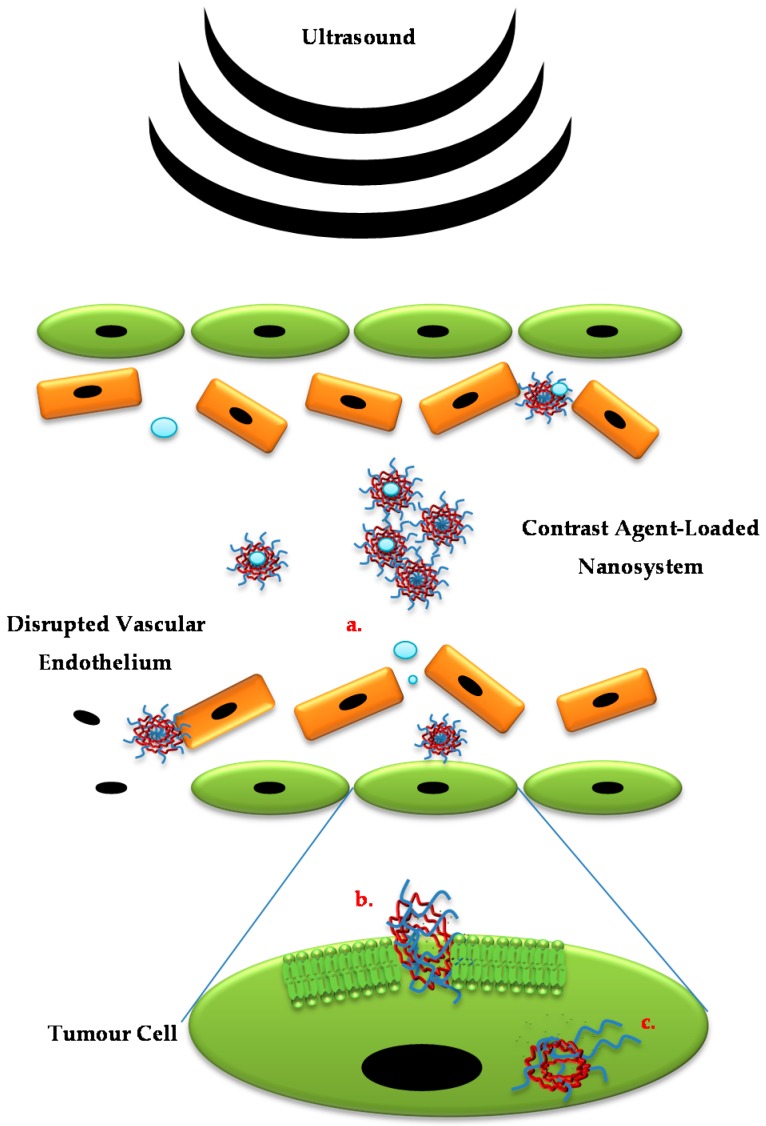
Schematic illustrating various ultrasound-responsive mechanisms: (**a**) Application of focused ultrasound guides nanosystems to the site of action and disrupts vascular endothelial barrier allowing the nanosystem direct access to tumour cells; (**b**) Sonoporation of the tumour cell membrane increases its permeability with possible sonochemical shape change of nanosystem promoting entry into the cell; (**c**) Should the nanosystem incorporate an ultrasound-responsive component, extracellular/intracellular nanosystem disruption with subsequent drug release is also promoted.

Gourevich and co-workers [93] undertook a study considering both the mechanical and thermal stimuli emanating from ultrasound and tested the stimuli individually on a doxorubicin γ-cyclodextran nanocarrier system (Figure 5) that was designed for the treatment of breast cancer. Each of these stimuli, i.e., mechanical, hyperthermia and focused ultrasound-induced heat, were compared to a control group and proved to cause an increase in encapsulated drug uptake. Doxorubicin cellular uptake was increased in the order of thermal application of focused ultrasound > hyperthermia without focused ultrasound > mechanical effects. Ultrasound caused drug release and sonoporation to assist with drug transfusion into the cells. Focused ultrasound (1.35 MHz) in conjunction with MRI guidance allowed the drug delivery process to be more efficient, safer, and accurate. Introduction of SonoVue (sulphur hexafloride) microbubbles permitted a significant increase in intracellular doxorubicin uptake [93] (Table 2). Ultrasound-responsiveness branches throughout a broad spectrum of stimuli allowing many other smart polymer systems to respond through heat production and chemical hydrolysis via a pH change. Figure 6 highlights various mechanisms of ultrasound-responsive chemotherapeutic delivery.

## 5. Pseudo-Ultrasound-Responsive Systems in Cancer Chemotherapeutics: Advancing Trends in Ultrasound-Responsive Systems

Due to the heat ultrasound generates during its application, thermoresponsive systems could also be employed in conjunction with ultrasound therapy as a complementary action. As highlighted in Table 2, Ninomiya and co-workers [94] designed Thermosensitive Liposomes (TSL) from *N*-isopropyl acrylamide and *N*-isopropyl methacrylamide whereby, upon exposure to locally-focused ultrasound (1 MHz, 30 s, 0.5 W/cm^2^), heat was produced which stimulated the TSL to release doxorubicin for the treatment of breast cancer.

Focused ultrasound employed in cancer therapy is useful due to its ability to initiate hyperthermia or ablative therapy for tumours. Yang and co-workers [95] designed a human atherosclerotic plaque-specific peptide-1 (HAP-1)-conjugated doxorubicin liposomal delivery system (HAP-1 Lipo-Dox) that operated synergistically with pulsed focused ultrasound for the treatment of brain tumours (glioblastoma). Results showed that pulsed HIFU reduced the tumour growth rate significantly in comparison to chemotherapy alone. The survival time in mice was significantly increased in mice treated with HAP-1 Lipo-Dox in conjunction with HIFU, compared to treatment with HAP-1 Lipo-Dox in the absence of sonication [95].

Pluronic is a well-known thermoresponsive triblock polymer (PEO-PPO-PEO) composed of basic poly(ethylene oxide) and poly(propylene oxide) units. A study was undertaken by Husseini et al. [96] using Pluronic P-105 micelles loaded with doxorubicin. An in vitro release test was undertaken upon continuous wave and pulsed ultrasound exposure to the micelles. Conclusive results proposed that cavitation played a vital role in the disturbance of the micelle structure that resulted in drug release of doxorubicin and upon withdrawal of the acoustic waves allowed the reformation of micelles and re-encapsulation of drug.

The delivery systems discussed above are stimulated by the “secondary” stimulus, i.e., heat generated via ultrasound, to temperatures high enough to activate drug release from these systems. Differentiating between whether the system responds to the thermal or ultrasound stimulus is important for future investigations for understanding and improvement of ultrasound-responsive drug delivery systems. Due to these thermoresponsive systems responding to ultrasound stimuli or being guided by ultrasound, they could be termed “pseudo-ultrasound”-responsive.

An interesting study was undertaken by Rapaport and co-workers [97]. Their group had synthesised paclitaxel-loaded Perfluorocarbon (PFC) nanodroplets in combination with a PEO-*co*-PLA block copolymer and employed these subsequently to the application of focused ultrasound for the treatment of pancreatic cancer. It was suggested that the time between injecting the formulation and applying the magnetic resonance imaging guidance focused ultrasound (MRgFUS) played a significant role in positive cancer treatment. This was due to the time that the formulation required to accumulate in the tumour tissue in order for an appropriate concentration of drug to be present for release prior to MRgFUS application. If this time is not considered, applying the ultrasound may lead to collateral damage to normal tissue [97].

## 6. An Overview of Various Chemotherapeutic Delivery Systems Combining Thermal- and Ultrasound-Responsive Polymers

A *N,N*-Diethylacrylamide (NNDEA) core Pluronic micellular solution (Plurogels^®^) and regular Pluronic P-105 micelles at low concentrations were formulated to release doxorubicin in response to low frequency ultrasound for synergistic therapy against leukaemia cells. The Plurogel, being stabilised in comparison to the unstable Pluronic micelles, provided enhanced protection of normal cells against the drug through micelle reformation and reuptake of drug into the micelle after removal of the ultrasound stimulus, leading to less exposure to the drug and lowering the possibility of side effects (Table 3) [111].

PLGA as well as Pluronic proved to be responsive to ultrasound [112]. According to Liu and co-workers [69], thermoresponsive micelles fabricated from the block copolymer, poly(*N*-isopropylacrylamide-*co*-*N,N*-dimethylacrylamide)-*b*-poly(d,l-lactide-*co*-glycolide) contained both thermoresponsive and ultrasound responsive components. In addition to PLGA’s superior biodegradability and drug-loading capacity, an increase in drug delivery could be achieved via application of ultrasound.

Polymethacrylate polymers are ionic polymers and are pH-responsive [23]. Research involving application of ultrasound by Wang and co-workers [113] has been undertaken with micelles assembled from a diblock coploymer of thermal- and pH-sensitive polymers. Their research demonstrated that applying HIFU to the micelles in an aqueous solution produced thermal and mechanical effects that initiated chemical hydrolysis that resulted in a decrease in pH and promoted drug release. This delivery system could be effective for tumoral delivery due to the lower pH ~5.7 [114] and higher temperatures of tumours than normal tissue, enabling the system to potentially respond to the natural tumour milieu. HIFU combined with this delivery system would be complementary and may have significant potential for tumour therapy.

**Figure 7:**
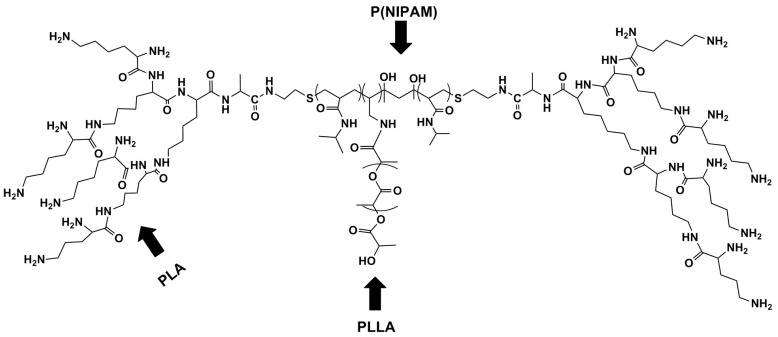
Structural configuration of sphingolipid ceramide loaded linear-dendritic nanoparticles grafted using P(NIPAM), PLLA and PLA [115]. (Source: Stover et al., 2008).

Stover and co-workers [115] developed sphingolipid ceramide loaded linear-dendritic nanoparticles by grafting P(*N*-Isopropyl Acrylamide)-P(Lactic Acid), [P(NIPAM)-PLA] with Poly-l-lysine-Poly-l-lactide (PLLA-PLA) (Figure 7) for apoptotic action in solid tumours, both of which contained PLA, that could be considered an ultrasound-responsive component. This system proved to be thermoresponsive and provided a long term sustained release of ceramide in the presence of hyperthermia [115]. This system therefore, could be triggered via a focused ultrasound stimulus as a source of hyperthermia.

A novel intravenous paclitaxel-loaded nano-emulsion was designed by Rapoport and co-workers [116], providing pancreatic and ovarian cancer chemotherapy through a conversion method technique from nano-emulsion to microbubbles via ultrasonic energy ranging between 90 KHz–1 MHz. The ultrasound-activated system containing paclitaxel decreased the tumour size significantly, whereas the nano-emulsion that did not contain paclitaxel had no effect on tumour shrinkage, which highlights that the effect was due to the ultrasound-enhanced chemotherapeutic action of the targeted paclitaxel, and not only the mechanical or thermal action of ultrasound. It was, however, reported that although tumour size reduction did occur, tumour resistance was an emerging factor and current research is being undertaken to eliminate resistance completely [116].

Another study undertaken demonstrated that the efficacy of ultrasound was dependent on the time allowed for accumulation of the drug at the site of the tumour subsequent to administration. This resulted in an increased uptake of drug into the tumour through micelle degradation or tumour perturbation [117]. Interestingly, even though the system partially comprised of Pluronic-105, ultrasound did not increase extravasation of the micelles even 30 min after the injection. It was further demonstrated that the mixed micelles retained 65% of drug in the core whereas standard Pluronic micelles released most of the drug encapsulated. It was indicated that these micelles are effective in the early stages of tumour appearance, however once the tumour has metastasized, surgical or ablative therapy should be considered before applying this delivery system.

It is reported that nano-sterically stabilised liposomes (nSSL) are known to be very efficient in tumours due to their “leakage” into the tumour [118]. A study of cisplatin-loaded nSSLs in the presence of low focused ultrasound was conducted for chemotherapy in colonic cancer in Albino Laboratory-bred (BALB/c) female mice. The cholesterol composition in these liposomes decreases the porosity as well as their sensitivity to heat. Using LFUS in the presence of drug loaded nSSLs, enhanced the therapeutic effect of delivery systems such as these, which do not release drug into the circulation [119]. According to Lin and co-workers [15] cholesterol and PEG-lipid content or lipid packing contributes greatly to ultrasound responsivity in a system. When the temperature increases, it inherently assists with the ultrasound-responsive sensitivity, rendering it challenging to distinguish the quantification of ultrasound- to thermo-sensitivity [120].

A dually-responsive ultrasound-pH responsive polymeric vesicle was synthesised as an antineoplastic delivery system. Poly ethylene oxide (PEO) is classified as thermoresponsive and poly(2-tetrahydrofuranoloxyethyl methacrylate), a pH-responsive polymer. As previously mentioned, ultrasound can activate thermoresponsive systems via heat production and stimulate pH-responsive systems via chemical reactions; thus overlapping heat and ultrasound. This novel polymeric vesicle proved to have the ability to encapsulate anticancer drugs, and release them at a controlled rate, being non-cytotoxic to liver cells below a concentration of 250 μg/mL but decreasing liver cell viability (by 25%) above 500 μg/mL. The system proved to successfully decrease liver cancer cell viability as ascertained via the CCK-8 in vitro quantification method [121].

A study undertaken by Howard et al. [122] was based on determining the effects of ultrasound on paclitaxel-loaded copolymeric micelles, designed from the thermoresponsive PEO, and PLA-tecopherol, where PLA is reported to be ultrasound-responsive in nature. When evaluated against breast tumour progression in combination with ultrasound (1 MHz), not only were normal cells protected from the toxic effects of the chemotherapeutic drugs, but the ultrasound assisted in increasing drug uptake by cancer cells, which was ascertained via in vitro studies, and increased tumour regression when tested in nude mice [122].

Micelles fabricated from PEO combined with different methacrylates were tested in response to HIFU, of which neither of the polymers is classified ultrasound-responsive. These micelles were encapsulated with nile red (NR) of lipophilic nature and upon exposure to HIFU, the micellular structure is disrupted through chemical hydrolysis and causes release of NR in a controlled manner [123]. Therefore, these micelles may be useful for loading and controlling the delivery of hydrophobic chemotherapeutic drugs.

A multifunctional nanoparticle injectable polymeric perfluorocarbon nanoemulsion comprising of a PEO-*b*-PLA diblock copolymer, a thermoresponsive and ultrasound-responsive classified combination, was synthesised to release encapsulated doxorubicin in response to therapeutic ultrasound to treat breast cancer. In conjunction with a diblock delivery system, drug-loaded, targeted nanobubbles were included, which expand to become microbubbles upon heating. Upon rupturing, cavitation occurs and ultimately intracellular drug uptake is enhanced; through these mechanisms chemotherapy was effectively undertaken [124]. Table 3 provides a summary of chemotherapeutic delivery systems with ultrasound and thermoresponsive attributes.

## 7. Conclusions and Future Perspective

An overview of thermoresponsive and ultrasound-responsive chemotherapeutic delivery systems was provided as a means of highlighting promising investigations for future innovative research directions in this field. Understanding the mechanisms of operation these drug delivery systems is essential for the advancement in stimuli-responsive system modification. Thermo-responsive delivery systems are widely developed and employed due to their efficient outcomes in drug delivery owing responsiveness to internal and external stimulation i.e., body temperature, and laser or ultrasound, respectively. In terms of external stimulation, drug delivery systems will evolve to an extent where most stimuli-responsive systems of various classes of smart polymers could be attuned to ultrasound-mediated delivery, but not necessarily be ultrasound-responsive. This is because ultrasound serves as a source of mechanical impulses that may simulate an environment similar to electric impulses, generating a change in heat to stimulate thermoresponsive delivery systems, and/or initiating chemical reactions in certain mediums to cause a pH change triggering drug release into the targeted site. Ultrasound, being a well-known safe diagnostic tool for biomedical applications such as imaging and therapy such as hyperthermia, could be used for many multi-responsive drug delivery systems. The application of ultrasound in futuristic approaches to drug delivery would extensively contribute to the enhancement of chemotherapeutic drug delivery. In stating that ultrasound induces mechanical effects, future research could branch into investigating the effect of ultrasound on thixotropic properties of drug delivery systems, and the responsivity to ultrasound and improvement in efficiency in the presence of ultrasound of chemotherapeutic delivery systems derived from intelligent materials other than ultrasound-, thermal- and pH-responsive materials.

## Figures and Tables

**Table 1:** Thermoresponsive systems in cancer therapy.

Thermoresponsive system	Bioactive	Cancer type	Switching temperature	Reference
P(NIPAM-*co*-AA)	Doxorubicin	Breast Cancer	5–50 °C	[48]
P(NIPAM)	Doxorubicin	Multimodal Cancer	33.4 °C, 38.3 °C	[53]
PEG-HAuNS	Doxorubicin	Breast Cancer		[54]
P(NIPAM-acrylamide-allylamine)	-	Prostate Cancer	40 °C	[55]
Chitosan-*g*-PEI	Bafilomycin Candesartan	Pancreatic Cancer	-	[66]
Elastin-Like peptide	Radionuclide	Advanced Stage Tumoral Cancers	27 °C, 62.5 °C	[67]
P(NIPAM-*co*-N,NDMAM), CHOL-*g*-P[NIPAM-*co*-*N*-(hydroxymethyl) acrylamide]	Indomethacin	-	-	[68]
P(NIPAM-*co*-N,NDMAM)-*b*-PLGA	Doxorubicin	Cancer	39.5 °C	[69]
P(NIMAM-*co*-N,NDMAM)-*b*-PLa	Adriamycin	Bovine Aorta Endothelial	40 °C	[70]
P(NIPAM-*co*-AAm)	No drug (rhodamine); Doxorubicin	Ovarian Cancer; Colon Cancer	40 °C, >0 °C	[71,72]
P(MOEGA–DMDEA)	Paclitaxel, Doxorubicin	-	23.8–35.1 °C	[73]
P(NIPAM-*co*-N,NDMAM)-*b*-P(LA-*co*-CL)	Doxorubicin	-	~40 °C	[74]
P(NIPAM-*b*-PBMA)	Adriamycin	-	32.5 °C	[75]
Pluronic F127–chitosan	Curcumin	Prostate Cancer	37 °C	[76]
(Fe_3_O_4_)-P(NIPAM)	Doxorubicin	Cancer	35 °C	[77]
PEG′d Liposomes	Doxorubicin	Liver Cancer	-	[78]
DPPC:DPPG:MSPC:mPEG_2000_-DSPE (HTLC)	Cisplatin	Cervical Cancer	40–43 °C	[79]
DPPC/MSPC/DSPE-PEG_2000_		Breast Cancer	37–42 °C	[80]
P(NIPAM)-NH_2_(Amino-terminated)	Adriamycin	-	32 °C	[81]
Pluronic 127	Paclitaxel, Docetaxel	Breast Cancer, Ovarian Cancer	>20 °C, 21 °C	[82,83]
DPPC:DSPC:DPTAP:DSPE:PEG_2000_	Doxorubicin	Lung Carcinoma	~39 °C	[84]
DPPC:MPPC:DPPE-PEG_2000_, DPPC:HSPC:CHOL:DPPE-PEG_2000_	[Gd(HPDO3A)(H_2_O)] + Doxorubicin	Squamous Cell Carcinoma	-	[85]
DPPC:CHOL:DSPE-PEG_2000_:DSPE-PEG_2000_-Folate	Doxorubicin	Epidermoid Cancer, Cervical Cancer	41.6 °C	[86]

**Table 2:** Polymeric drug delivery systems responsive to ultrasound in cancer therapy.

Ultrasound-Responsive system	Bioactive	Cancer type	Reference
PLGA	Methotrexate	Placental Chariocarcinoma	[87]
PMMA	Doxorubicin	Liver Cancer	[92]
γCyclodextran-*N*-Boc-3-(2-naphthyl)-alanine	Doxorubicin	Breast Cancer	[93]
APT:P(NIPAM-*co*-NIPAM)	Doxorubicin	Breast Cancer	[94]
l-α-phosphatidylcholine:CHOLl:DSPE-PEG_2000_	Doxorubicin	Glioblastoma	[95]
PDA:PDLA-PFC	Paclitaxel	Pancreatic Cancer	[97]
Pluronic	Doxorubicin	-	[96]
(DSPE-PEG_2000_-Biotin)	Paclitaxel	Breast Cancer	[98]
DSPC:DSPE-PEG_2000_:DSPG	Doxorubicin	Brain Cancer	[99]
DPPC:DPSE:PEG_2000_:Biotin	1,3-bis(2-chloroethyl)-1-nitrosourea	Glioma (Brain/Spinal Tumor)	[100]
CHC:SPIO:BSA (MB)	Camptothecan	Breast Tumour Cells	[101]
DPPC-DSPE-PEG-maleimide:CHOL	Doxorubicin	Lung Cancer	[102]
PLA	Paclitaxel, Doxorubicin	Breast Cancer, Liver Cancer	[103,104]
DPPC:DPPG:DPPE-PEG_2000_	Doxorubicin	Cancer	[105]
DPPC:DPPA:DPPE-PEG_2000_	10-Hydroxycamptothecin	Liver Cancer	[106]
Lysolecithin-containing DPPC:MSPC:DSPE-PEG_2000_	Doxorubicin	Squamous Cell Carcinoma	[107]
DPPC:MSPC:DSPE-PEG_2000_	TO-PRO-3 (DNA-intercalating agent)	Glioma	[108]
DSPC: DSPE-PEG(2k)-OMe	HLA (Antigen)	-	[109]
DSPC: DSPE-PEG(2k)-OMe	IL-12	Ovarian Cancer	[110]

**Table 3:** Delivery systems incorporating thermo- and ultrasound-responsive components.

Thermoresponsive component	Ultrasound-Responsive component	Bioactive	Cancer type	References
N,N-DEAM	Pluronic	Doxorubicin	Leukaemia (HL-60)	[111]
P(NIPAM)-*co*-N,NDMAM	PLGA	Doxorubicin	Breast Cancer	[112]
PEO	Poly(THPMA)	-	-	[113]
P(NIPAM)-PLA	PLLA-PLA	Sphingolipid ceramide	Cancer	[115]
PEO	PLA	Paclitaxel	Pancreatic, Ovarian, Breast Cancer Tumours	[116]
PEG_2000_-diacylphospholipid	Pluronic P-105	Doxorubicin	Ovarian Cancer	[117]
PEG	P(βA)	Doxorubicin	Ovarian Cancer	[117]
PE: DSPE	CHOL	Cisplatin	Colon Cancer	[119]
DPPC	(DPPE-PEG_2000_)	Calcein	-	[120]
PEO	P(2-THFMA)	Doxorubicin	Liver Cancer	[121]
PEO	PLA-tocopherol	Paclitaxel	Breast Cancer	[122]
P(NIPAM)	PLA	-	-	[123]
PEO, P(NIPAM)	2-hydroxymethyl methacrylate	Doxorubicin	-	[124]
PEO	PIBMA, P(2-THMA)	-	-	[125]
PEO	PLA, PCL	Doxorubicin	Breast Cancer	[126]
P(NIPAM-*co*-PAA)	Propylacrylic acid	Doxorubicin	Breast Cancer	[127]

## Abbreviations

**Abbreviation**	**Compound Name**
AA	Acrylic Acid
AAm	Acrylamide
PBMA	Poly(Butyl Methacrylate)
BSA	Bovine Serum Albumin
CHC	Carboxymethyl-Hexanoyl Chitosan
CHOL	Cholesterol
DMDEA	2-(5,5-Dimethyl-1,3-Dioxan-2 yloxy) Ethyl Acrylate
DPPC	1,2-Dipalmitoyl-*sn*-glycero-3-Phosphatidylcholine
DPPE	Dipalmitoyl-*sn*-glycero-3-Phosphatidylethanolamine
DPPG	Dipalmitoyl Phosphatidylglycerol
DPSC	1,2-Distearoyl-*sn*-glycero-Phosphcholine
DPSE	1,2 Distearoyl-*sn*-glycero-3-Phosphoethanolamine
DSPC	1,2-Distearoyl-*sn*-glycero-3-Phosphotidylcholine
DSPE	1,2-Distearoyl-*sn*-glycero-3-Phosphoethanolamine
DSPG	1,2-Dioctadecanoyl-*sn*-glycero-3-Phospho-(1′-rac-glycerol)
HAP-1	Human Atherosclerotic Peptide-1
HMM	2-Hydroxymethyl Methacrylate
MOEGA	Monomethyl Oligo (Ethylene Glycol) Acrylate
MSPC	1-stearoyl-2-hydroxy-*sn*-glycero-3-phosphocholine
N-DMAM	*N*-Dimethylacrylamide
N-(HM)A	*N*-Hydroxymethyl Acrylamide
N′N DMAM	*N,N*-Dimethylacrylamide
P(βA)	Poly(β Benzyl-l-Aspartate)
P(THFMA)	Poly(2-Tetrahydrofuranoloxy) Ethylmethacrylate
P(THPMA)	Poly(2-Tetrahydropyranyl) Methacrylate
PCL	Poly-ε-Caprolactone
PEG	Polyethylene Glycol
PEG'd	Polyethylene Glycolated
PEI	Polyethyleneamine
PEO	Polyethylene Oxide
PIBMA	Poly[(Isobutoxy) Ethyl Methacrylate]
PFC	Perfluorocarbon
PFH	Perfluorohexane
PFP	Perfluoropentane
PLA	Poly(Lactide)/Poly(Lactic Acid)
PLGA	Poly(d,l-Lactide-*co*-Glycolide)
PLLA	Pol-l-Lysine
PMA	Poly(Methacrylate)
PMMA	Poly(Methyl Methacrylate)
P(NIPAM)	Poly(*N*-Isopropyl Acrylamide)
SPIO	Super Paramagnetic Iron Oxide
